# The association between first-time accreditation and the delivery of recommended care: a before and after study in the Faroe Islands

**DOI:** 10.1186/s12913-021-06952-w

**Published:** 2021-09-05

**Authors:** Maria Daniella Bergholt, Anne Mette Falstie-Jensen, Peter Hibbert, Barbara Joensen Eysturoy, Gunnvá Guttesen, Tóra Róin, Jan Brink Valentin, Jeffrey Braithwaite, Christian von Plessen, Søren Paaske Johnsen

**Affiliations:** 1 Department of Clinical Epidemiology, Aarhus University Hospital, Oluf Palmes Allé 43-45, DK-8200 Aarhus N, Denmark; 2 Department of Anesthesiology and Intensive Care Medicine, Copenhagen University Hospital, Gentofte Hospital, Gentofte Hospitalsvej 1, DK-2900 Hellerup, Denmark; 3 Danish Clinical Registries (RKKP), Oluf Palmes Allé 15, DK-8200 Aarhus N, Denmark; 4 Centre for Healthcare Resilience and Implementation Science, Australian Institute of Health Innovation, Macquarie University, Level 6, 75 Talavera Rd, Sydney, New South Wales 2109 Australia; 5 Australian Centre for Precision Health, University of South Australia Cancer Research Institute (UniSA CRI), School of Health Sciences, University of South Australia, GPO Box 2471, Adelaide, SA 5001 Australia; 6 Surgical Centre, National Hospital of the Faroe Islands, J.C Svabosgøta 41-49, 100, Tórshavn, Faroe Islands; 7 Medical Centre, National Hospital of the Faroe Islands, J.C Svabosgøta 41-49, 100, Tórshavn, Faroe Islands; 8 Department of Quality Improvement and Patient Safety, National Hospital of the Faroe Islands, J.C Svabosgøta 41-49, 100, Tórshavn, Faroe Islands; 9 Danish Center for Clinical Health Services Research, Department of Clinical Medicine, Aalborg University and Aalborg University Hospital, Frederik Bajers vej 5, DK-9220 Aalborg, Denmark; 10 Unisanté, Rue du Bugnon 44, CH-1011 Lausanne, Switzerland; 11 Institute for Clinical Research, University of Southern Denmark, Campusvej 55, DK-5230 Odense M, Denmark

**Keywords:** Accreditation, Hospital, Recommended care, Before and after study, Medical record audit

## Abstract

**Background:**

Significant resources are spent on hospital accreditation worldwide. However, documentation of the effects of accreditation on processes, quality of care and outcomes in healthcare remain scarce. This study aimed to examine changes in the delivery of patient care in accordance with clinical guidelines (recommended care) after first-time accreditation in a care setting not previously exposed to systematic quality improvement initiatives.

**Methods:**

We conducted a before and after study based on medical record reviews in connection with introducing first-time accreditation. We included patients with stroke/transient ischemic attack, bleeding gastric ulcer, diabetes, chronic obstructive pulmonary disease (COPD), childbirth, heart failure and hip fracture treated at public, non-psychiatric Faroese hospitals during 2012–2013 (before accreditation) or 2017–2018 (after accreditation). The intervention was the implementation of a modified second version of The Danish Healthcare Quality Program (DDKM) from 2014 to 2016 including an on-site accreditation survey in the Faroese hospitals. Recommended care was assessed using 63 disease specific patient level process performance measures in seven clinical conditions. We calculated the fulfillment and changes in the opportunity-based composite score and the all-or-none score.

**Results:**

We included 867 patient pathways (536 before and 331 after). After accreditation, the total opportunity-based composite score was marginally higher though the change did not reach statistical significance (adjusted percentage point difference (%): 4.4%; 95% CI: − 0.7 to 9.6). At disease level, patients with stroke/transient ischemic attack, bleeding gastric ulcer, COPD and childbirth received a higher proportion of recommended care after accreditation. No difference was found for heart failure and diabetes. Hip fracture received less recommended care after accreditation. The total all-or-none score, which is the probability of a patient receiving all recommended care, was significantly higher after accreditation (adjusted relative risk (RR): 2.32; 95% CI: 2.03 to 2.67). The improvement was particularly strong for patients with COPD (RR: 16.22; 95% CI: 14.54 to 18.10).

**Conclusion:**

Hospitals were in general more likely to provide recommended care after first-time accreditation.

**Supplementary Information:**

The online version contains supplementary material available at 10.1186/s12913-021-06952-w.

## Background

Recent decades have seen substantial advances in patients receiving safe and high-quality healthcare [1–5]. The introduction of evidence-based medicine [6] in combination with systematic quality improvement initiatives [7], including accreditation [8], have played a central role in the efforts to ensure that patients receive the best possible care and achieve the best possible outcome [9–11].

Accreditation is an external review process to assess how well an organization performs relative to established organizational and patient related standards [12]. Accreditation was established more than a century ago and has since become a widely adopted intervention [13]. Today more than 100 countries use accreditation as an important element in their quality improvement strategy [14]. Despite its popularity, the effectiveness of accreditation is often debated due to perceptions that it can be bureaucratic and time-consuming, and uncertain evidence as to its efficacy. Past research on accreditation has been criticized for methodological limitations and inconsistent results [15–21]. Hence, there is a need for more robust empirical research into the effectiveness of accreditation to determine its value [22–24]. Accreditation should ideally be studied in a setting not exposed to other systematic quality improvement initiatives to examine how and to what extent it affects patient care. This unique setting was present in the Faroe Islands before its first hospital accreditation in 2017.

This study therefore aimed to examine accreditation-related changes in the delivery of care in accordance with clinical guidelines (recommended care) in connection with first-time hospital accreditation on the Faroe Islands. Based on past research, we hypothesized that accreditation would be associated with increased adherence to recommended care.

## Methods

### Study design

We conducted a before and after study on the delivery of recommended care for seven clinical conditions representing both acute and chronic diseases in relation to the first-time accreditation of the Faroe Islands hospitals.

### Setting

The Faroe Islands consists of 18 islands in the North Atlantic. It is an autonomous territory within the Kingdom of Denmark. The Faroe Islands are classified as a high-income country by the World Bank (GDP per capita, 2016, USD$ 55,823) [25] with a population of 52,584 people [26] predominantly of Scandinavian descent. The healthcare system is financed through taxation and all hospital healthcare is free of charge. The Faroe Islands three hospitals, The National Hospital in the capital Torshavn, Klaksvik Hospital and Suderø Hospital have never participated in accreditation or other systematic quality improvement activities before the first hospital accreditation in February 2017.

### Intervention

The three hospitals were assessed for accreditation through an on-site survey in February 2017 by the Danish Institute for Quality and Accreditation in Healthcare (IKAS) using the second version of the Danish Healthcare Quality program (DDKM) [27], modified for the Faroese Healthcare system [28]. IKAS had modified the 76 hospital standards in consultation with stakeholders in the Faroese health care system ensuring all standards being aligned with Faroese legislation.

All hospitals participated voluntarily in the first accreditation program. Updating existing policies, instructions and guidelines as well as developing entirely new evidence-based ones was a high priority throughout the implementation process from 2014 to 2016. All new documents were placed in a new electronic document management system ensuring all health professionals access to the latest and updated version wherever they were in the hospital. In addition, much time was spent implementing new workflows and teaching staff all new initiatives. In parallel with the implementation process, an IT system for recording adverse events was developed. In addition, work began systematically on an electronic patient system that contained all patient data, ensuring all patient information was in one place and accessible to all healthcare professionals. During the on-site survey in February 2017, a team of surveyors assessed compliance with the standards through observation, interviews and review of the hospital documentation [9]. All three hospitals were subjected to the on-site survey the same week. The Danish Accreditation Award Committee subsequently awarded Klaksvik hospital full accreditation. Suderø hospital and the National hospital were not initially fully compliant with the accreditation standards but were after a follow-up survey (an interview after submitting additional documentation) assigned full accreditation in May and September 2017 respectively.

### Recommended care

We measured the ability of hospitals to deliver recommended care using the disease specific process performance measures from the National Clinical Registries. Each year, the level of quality of care delivered to patients in Danish healthcare, is evaluated at national level for each disease area. Based on the evaluations and the current evidence, all requirement regarding each disease specific process performance measure are updated. In the present study we used the requirements related to the year 2016 [29]. All measures was developed by expert panels in The Danish Clinical Quality Program – National Clinical Registries (RKKP) [29–34]. The process performance measures, and time limits included in the study all reflect recommendations from national clinical guidelines. However, not all process performance measures in the national registries were relevant, as some specialized treatments were not available in the Faroe Islands. Therefore, we chose 63 relevant disease specific process performance measures for seven clinical conditions. Stroke and transient ischemic attack (stroke/TIA) (12 measures), bleeding gastric ulcer (8 measures), diabetes (12 measures), COPD (11 measures), childbirth (3 measures), heart failure (7 measures) and hip fracture (10 measures). All process performance measures with time frames and diagnosis codes are provided in Additional file 1.

### Participants

We assessed eligibility for patients through the Faroese National patient register. The register holds information about all patients treated in the Faroese healthcare system. In- and outpatients with one of seven clinical conditions, were eligible for inclusion if they were ≥ 18 years (≥ 30 years for patients with COPD) and had been treated in one of the three hospitals during 2012 and 2013 (before accreditation) or during 2017 and 2018 (after accreditation). Due to different accreditation dates, patients from Klaksvik, Suderø and the National hospital were included after February 21, June 1, September 20, 2017 respectively. Diabetics were only included as outpatients. Patients with COPD were included as in- and outpatients. All other groups only included inpatients. Patients with multiple hospital contacts (with the same clinical condition) were only included with their first appearance in the study period. Patients treated for different clinical conditions were included once for each condition, as inclusion for one condition was considered independent of the others.

**Fig. 1:**
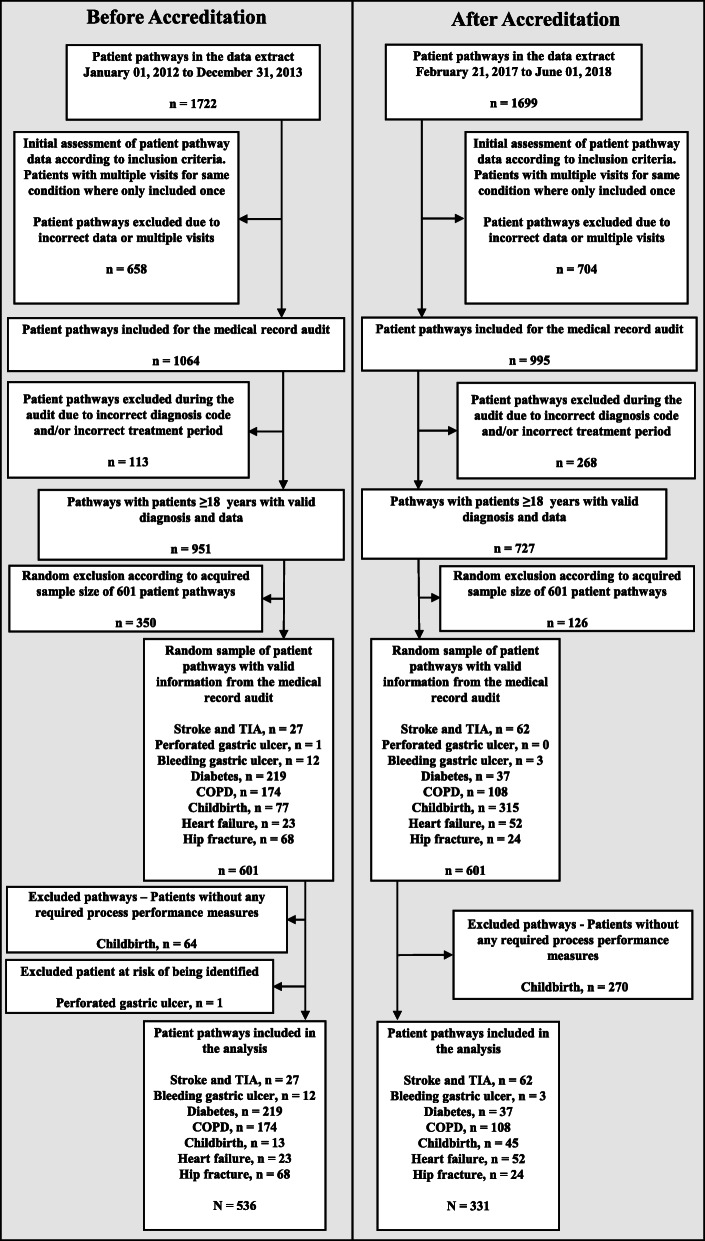
Flowchart on patient pathway before and after the first accreditation

The registers included a total of 1722 patient pathways before and 1699 patient pathways after accreditation. Of these, we excluded respectively 835 patient pathways before accreditation and 1242 patient pathways after accreditation due to mismatches between recorded diagnosis and the true reason for a hospitalization/outpatient visit, incorrect treatment period, multiple visits and/or incomplete documentation of process performance measures. For more details, see Fig. 1.

### Data collection

We developed a medical record audit tool and database using REDCap [35]. Two medical auditors retrieved data before accreditation and four after accreditation. One of the medical auditors participated in both data collections to ensure consistency. The medical auditors were all Faroese with local contextual knowledge, and all had a bachelor’s degree in medicine.

Data on recommended care were obtained through electronic and paper medical records. The medical auditors initially screened the medical records for inclusion criteria. Recommended care was registered in four categories as: “yes”, care provided was consistent with the measure;, “no”, care provided was not consistent with the measure;, “unknown”, no data in the medical record related to the measure; and “not applicable” i.e., the measure was not relevant for the patient. All data were double-checked, and to ensure reliability, two auditors independently entered data from 100 randomly chosen patients; Cohen’s kappa = 0.86 [36].

For a power of at least 80% and a Z-alpha value of 1.96, 601 medical records were needed before and after the first accreditation, respectively for detecting differences in the relative risk of receiving recommended care of 1.2. We estimated the chance of receiving recommended care per encounter before accreditation to be 40%.

### Statistical analysis

Initially, we conducted a descriptive summary of baseline characteristics stratified by before and after accreditation, presenting categorical variables as frequencies and percentages and continuous variables as means and ranges.

For the primary analyses, the level of recommended care was analyzed as an opportunity-based composite score and an all-or-none score. All scores involved the fulfillment of individual process performance measures.

The opportunity-based composite score [37] reflected the proportion of fulfilled eligible process performance measures. The all-or-none score reflected the number of patient pathways who had received care fulfilling all relevant process performance measures. Effect measures comparing the period after accreditation with the period before were presented as percentage point difference for the opportunity-based composite score and as relative risk (RR) and risk difference (RD) for the all-or-none score. The analyses were conducted on a total score including all clinical conditions and stratified by clinical conditions. The all-or-none analyses were restricted to patients with a minimum of two relevant process performance measures. A sensitivity analysis was subsequently undertaken including all patients. In addition, we estimated RR for each individual indicator, which were presented in a forest plot.

We computed the RR using Poisson regression with robust variance. The percent point difference as well as the RD was calculated using linear regression. In all cases, we used mixed effects analyses with a random intercept at patient- and hospital level to account for recurrent patient dependence as well as within hospital dependence. When the models were unable to converge, we used the clustered sandwich estimator for the patient-level or patient-level dependence was ignored. A two-sided significance level of 5% was applied. Data were analyzed in StataSE, version 14.2. (StataCorp, 2015. College Station, TX: StataCorp LLC).

## Results

A total of 867 patient pathways with 6023 relevant process performance measures were included in the analysis, corresponding to 536 before and 331 patient pathways after accreditation with 4284 and 1739 relevant measures, respectively. A total of 9 patients before accreditation and 40 patients after accreditation were treated more than once for different clinical conditions at the Faroese hospitals. Before and after accreditation the average age of patients was 66 years. More women were included after accreditation (44.0% vs 55.6%). The proportion of inpatients was higher after accreditation (39.7% vs 79.2%) and more often admitted to specialist departments after accreditation (13.6% vs 67.6%). After accreditation, more patients were hospitalized with stroke and TIA (5.0% vs 18.7%), childbirth (2.4% vs 13.6%) and heart failure (4.3% vs 15.7%) (Table 1).

**Table 1:** Patient pathway characteristics

Characteristic	Before Accreditation 2012 and 2013	After Accreditation 2017 and 2018
	*N* = 536	*N* = 331
Sex, n (%)		
Male	300 (56.0)	147 (44.4)
Female	236 (44.0)	184 (55.6)
Age, n (%)		
< 50 years	98 (18.3)	71 (21.4)
50–75 years	282 (52.6)	135 (40.8)
> 75 years	156 (29.1)	125 (37.8)
Age (years)		
Mean (range)	66 (18–97)	66 (18–96)
Cohabitant status, n (%)		
Cohabitant	288 (53.7)	193 (58.3)
Living alone	84 (15.7)	49 (14.8)
Other, i.e. Nursing home	44 (8.2)	35 (10.6)
Undisclosed	120 (22.4)	54 (16.3)
Employment status, n (%)		
Working	133 (24.8)	63 (19.0)
Not working e.g. Retirees	178 (33.2)	99 (29.9)
Undisclosed	225 (42.0)	169 (51.1)
Type of admission, n (%)		
Inpatient	213 (39.7)	262 (79.2)
Outpatient	323 (60.3)	69 (20.8)
Type of inpatient, n (%)		
Acute	206 (96.7)	248 (94.7)
Scheduled	7 (3.3)	14 (5.3)
Inpatient department, n (%)		
Surgical	65 (30.5)	27 (10.3)
Medical	92 (43.2)	8 (3.0)
Mixed (surgical/medical)	27 (12.7)	50 (19.1)
Specialist e.g. Cardiology	29 (13.6)	177 (67.6)
Treating hospital, n (%)		
The National hospital	449 (83.8)	257 (77.6)
Klaksvik hospital	55 (10.3)	47 (14.2)
Suderø hospital	32 (5.9)	27 (8.2)
Clinical conditions, n (%)		
Stroke and Transient ischemic attack	27 (5.0)	62 (18.7)
Bleeding gastric ulcer	12 (2.2)	3 (0.9)
Diabetes	219 (40.9)	37 (11.2)
Chronic obstructive pulmonary disease	174 (32.5)	108 (32.6)
Childbirth	13 (2.4)	45 (13.6)
Heart failure	23 (4.3)	52 (15.7)
Hip fracture	68 (12.7)	24 (7.3)

### Changes in opportunity-based composite scores

The total opportunity-based composite score was higher after accreditation (adjusted percentage point difference (%): 4.4%; 95% CI: −0.7 to 9.6) but the difference did not reach statistical significance. The largest difference was found for childbirths that received 27.9% (95% CI: 24.8 to 31.0) more recommended care after accreditation. Patients treated for stroke/TIA, bleeding gastric ulcer and COPD had a difference of respectively, 17.6% (95% CI: 9.7 to 25.4), 22.5% (95% CI: 18.9 to 26.2) and 14.3% (95% CI: 5.5 to 23.1) after accreditation. No significant differences were found for patients with heart failure. In contrast, patients with diabetes and hip fractures received less recommended care after accreditation with a difference of − 4.3% (95% CI: − 6.2 to − 2.4) and − 5.9% (95% CI: − 8.7 to − 3.1) (Table 2).

**Table 2:** The opportunity-based composite score according to clinical condition before and after the first accreditation

	Before Accreditation 2012 and 2013			After Accreditation 2017 and 2018				
	N	Unadjusted Mean (%)	(95% CI)^a^	N	Unadjusted Mean (%)	(95% CI)	Adjusted Difference (%)^b^	(95% CI)
Clinical condition								
Stroke and TIA	27	50.9	(39.9;62.5)	62	69.7	(62.6;76.7)	17.6	(9.7;25.4)
Bleeding gastric ulcer	12	36.7	(26.9;46.5)	3	58.3	(14.7;100)	22.5	(18.9;26.2)
Diabetes	219	70.8	(68.2;73.4)	37	68.2	(61.0;75.3)	−4.3	(−6.2; −2.4)
COPD	174	15.5	(11.8;19.2)	108	25.3	(18.0;32.6)	14.3	(5.5;23.1)
Childbirth	13	10.2	(0.0;27.4)	45	38.1	(27.6;48.6)	27.9	(24.8;31.0)
Heart failure	23	59.5	(45.5;72.5)	52	56.1	(48.2;64.0)	−1.2	(−4.2;1.7)
Hip fracture	68	34.4	(31.0;37.8)	24	27.7	(23.6;31.9)	−5.9	(−8.7; −3.1)
Total	536	44.6	(41.8;47.4)	331	45.0	(41.6;49.4)	4.4	(−0.7;9.6)

^a^*CI* Confidence interval ^b^Adjusted for dependence between observations at patient level and cluster effect at hospital level

### Changes in all-or-none scores

The all-or-none score for all clinical conditions was statistically significant higher after accreditation (adjusted relative risk (RR): 2.32; 95% CI: 2.03 to 2.67). At condition levels, patients with COPD were more likely to receive all the recommended care after accreditation (RR: 16.22; 95% CI: 14.54 to 18.10). The results were unchanged for patients with stroke/TIA and diabetes. In contrast, patients with heart failure were less likely to receive recommended care after accreditation, (RR: 0.44; 95% CI: 0.29 to 0.66) however the risk difference (RD) was not statistically significant, (RD: -0.12; 95% CI: − 0.25 to 0.01) (Table 3). Overall results remained the same when including all patients with no restrictions on the number of included process performance measures in a sensitivity analysis. However, the relative risk for receiving all the recommended care increased significantly for childbirth (RR: 2.59; 95% CI: 1.93 to 3.49) (see Additional file 2).

**Table 3:** The proportion of patient pathways who received 100% of the recommended care before and after the first hospital accreditation

	Before Accreditation 2012 and 2013		After Accreditation 2017 and 2018			
	All recommended care (N)^a^	All recommended care (%)	All recommended care (N)	All recommended care (%)	RR^b^ (95% CI)^c^	RD^d^ (95% CI)
Clinical condition						
Stroke and TIA	2/27	7.4	17/62	27.4	3.69 (0.76;17.91)	0.20 (−0.01;0.41)
Bleeding gastric ulcer	0/12	0.0	0/3	0.0	–	–
Diabetes	17/219	7.8	3/37	8.1	1.04 (0.84;1.29)	0.003 (− 0.013;0.019)
COPD	1/104	1.0	5/32	15.6	16.22 (14.54;18.10)	0.147 (0.146;0.148)
Childbirth	0/12	0.0	0/30	0.0	–	–
Heart failure	5/23	21.7	5/52	9.6	0.44 (0.29;0.66)	-0.12 (−0.25;0.01)
Hip fracture	0/68	0.0	0/24	0.0	–	–
Total all-or-none	25/465	5.4	30/240	12.5	2.32 (2.03;2.67)	0.07^e^ (0.05;0.09)

^a^Number of patient pathways who received 100% of the recommended care according to the clinical condition divided by the number of patient pathways eligible for the care. All patients have a minimum of two relevant process performance measures. ^b^*RR* Relative Risk. Adjusted for dependence between observations at patient level and cluster effect at hospital level. ^c^*CI* Confidence interval. ^d^*RD* Risk difference. Adjusted for cluster effect at hospital level. ^e^Adjusted for dependence between observations at patient level and cluster effect at hospital level

### Changes in individual process performance measures

**Fig. 2:**
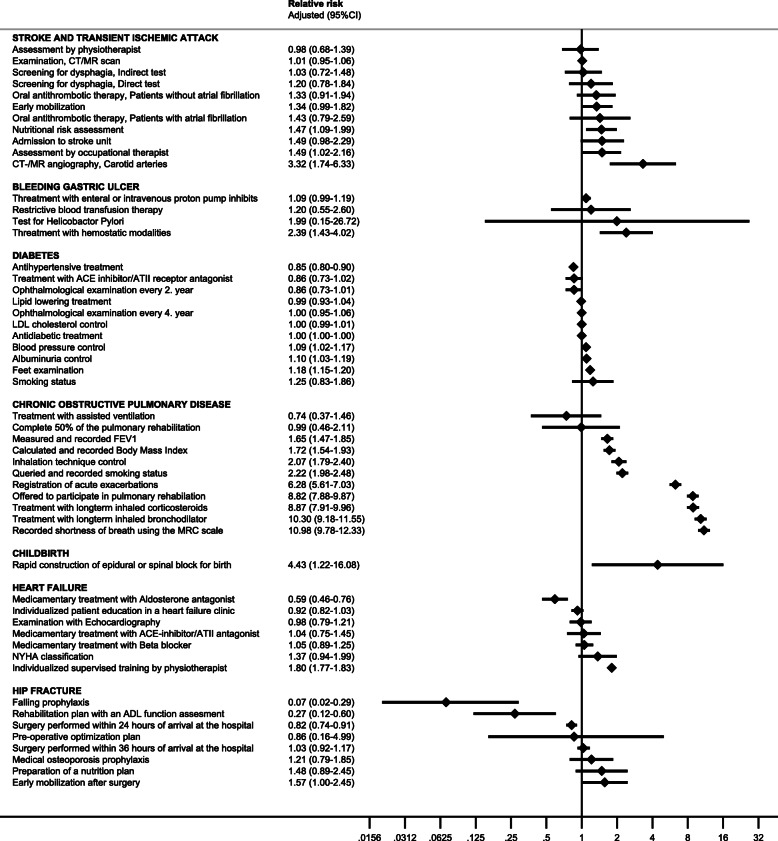
The probability of receiving a process performance measures according to clinical condition after first-time accreditation. The relative risk estimates are adjusted for dependence between observations at patient level and cluster effect at hospital level

Based on the calculated process performance measures (Fig. 2) a total of 19 process performance measures improved, 29 stayed unchanged and 5 declined. Overall, patients with COPD were found to have the greatest improvements after accreditation. A total of nine individual COPD process performance measures improved. Notably, the use of the Medical Research Council shortness of breath scale (RR: 10.98; 95% CI: 9.78 to 12.33), treatment with long-term inhaled bronchodilators (RR: 10.30; 95% CI: 9.18 to 11.55), long-term inhaled corticosteroids (RR: 8.87; 95% CI: 7.91 to 9.96) and participation in pulmonary rehabilitation (RR: 8.82; 95% CI: 7.88 to 9.87) improved significantly. Treatment with assisted ventilation and completing a pulmonary rehabilitation remained unchanged after accreditation. Mothers during childbirth were also significantly more likely to timely receive an epidural or spinal block after accreditation (RR: 4.43; 95% CI: 1.22 to 16.08). Patients with stroke were more likely to be assessed by an occupational therapist (RR: 1.49; 95% CI: 1.02 to 2.16) and by a nutritionist after accreditation (RR: 1.47; 95% CI: 1.09 to 1.99). The greatest improvement was observed for CT/MR angiography in patients with stroke and TIA (RR: 3.32; 95% CI: 1.74 to 6.33). For diabetics, the probability of having a foot examination (RR: 1.18; 95% CI: 1.15 to 1.20), an albuminuria (RR: 1.10; 95% CI 1.03 to 1.19) and blood pressure control (RR: 1.09; 95% CI: 1.02 to 1.17) improved significantly after accreditation. In contrast, the probability of receiving antihypertensive treatment declined after accreditation (RR: 0.85; 95% CI: 0.80 to 0.90). For patients with bleeding gastric ulcer, hemostatic treatment improved (RR: 2.39; 95% CI: 1.43 to 4.02). Patients with heart failure had a greater chance of receiving supervised physical mobilization during their hospitalization (RR: 1.80; 95% CI: 1.77 to 1.83). Yet, the likelihood of being treated with an aldosterone antagonist was lower after accreditation (RR: 0.59; 95% CI: 0.46 to 0.76). Patients with hip fracture had a greater chance of post-surgery mobilization after accreditation (RR: 1.57; 95% CI: 1.00 to 4.45), however the chances of surgery within 24 h of admission (RR: 0.82; 95% CI: 0.74 to 0.91), a rehabilitation plan (RR: 0.27; 95% CI: 0.12 to 0.60) or fall prophylaxis (RR: 0.07; 95% CI: 0.02 to 0.29) were lower after accreditation (Fig. 2).

## Discussion

To our knowledge, this is the first before and after study of voluntary hospital accreditation in a setting not previously exposed to any systematic quality improvement initiatives. The unique context offered by the Faroe Islands made it possible to examine this intervention in significant detail. Following first-time accreditation of the Faroe Islands hospitals, hospitals were in general more likely to provide recommended care to patients. The improvement was most evident when the level of care meets all process performance measures, reflecting ‘perfect care’ (all-or-none). Echoing our findings, a study of U.S. critical access hospitals including 45 states, found that accredited hospitals more often provided their patients with recommended care [38]. Similarly, a recent Danish study [9] found hospitals with high compliance with accreditation standards, were more likely to deliver recommended care in hospitals. Although, the cause and effect relationship in the US study could not be determined, due to the cross-sectional design, these results indicate that accreditation of hospitals is associated with more guideline adherent care and hence improved quality of care to patients in hospitals [39]. In contrast, another Danish study [17] found no difference between accredited and non-accredited hospitals in the delivery of recommended care. Indeed, non-accredited hospitals outperformed accredited hospitals in the overall opportunity-based composite score. There are several possible explanations for these conflicting findings. The hospitals in Denmark and the Faroe Islands were accredited by different accreditation programs. Furthermore, hospitals in Denmark had for several years been subjected to many different quality and safety activities; this could have led to the establishment of high levels of quality of care before introducing accreditation. If so, higher levels of care may have been difficult to improve in Denmark using accreditation [40].

Improvement was, however, not found for all clinical conditions in our study. For instance, the overall quality of care for diabetes, heart failure and hip fracture did not benefit from accreditation. For diabetes, the proportion of patients receiving care in accordance with the process performance measures was already high before accreditation and therefore difficult to improve. These results are consistent with other studies [41, 42]. The unchanged heart failure care and reduced levels of recommended care to patients with hip fractures was surprising, as pre-existing levels of care were below those found in similar studies [9, 17]. One explanation could be that treating physicians might have considered some of the recommended care not applicable for the patient, however such a decision should have been documented in the medical record. Another explanation could be that accreditation does not affect the delivery of recommended care in all clinical conditions the same way and at the same speed. Similar results were found in a study from Saudi Arabia [43].

Patients with COPD received significantly more recommended care following first-time accreditation in our study. It is not clear why there was such a substantial improvement, but the recruitment of a specialist in respiratory medicine, employed at the National Hospital in 2016 could explain a part of the progress, however a single specialist being able to raise the quality of treatment so markedly for all COPD patients in all hospitals over a short period seems unrealistic. Also, patients hospitalized with pulmonary diseases were after accreditation moved from a general medical department to a specialized department for heart and lung patients. We cannot know whether this reorganization have affected the delivery of recommended care to COPD patients. Regardless, the improvement is important for this patient group, as they often entail higher socioeconomic costs and in general have a poor survival rate compared to those with many other clinical conditions [43–45]. We also found, when viewing all seven clinical conditions combined, that patients had a greater chance of receiving all recommended care after accreditation. An improvement in the all-or-none score is a great achievement for any hospital. High scores often emphasize that a hospital can handle the most challenging care problems [46]. Existing literature has found that improvements in all-or-none scores are associated with better patient outcomes [47, 48].

Interestingly, the overall level of recommended care before and after accreditation was below 50% which is lower than in other countries [9, 49, 50]. This may be explained by several factors, including a possible lack of specialized doctors, monitoring, no systematic quality improvement activities, and minimal transparency related to the level of care delivered in the Faroese hospitals. Hence, it is difficult to foster improvements if clinicians have no or very little knowledge about the levels of care being delivered. In such a context, it is not surprising that the overall effects of the first-time accreditation were modest. More profound changes and repeated cycles of accreditation and other quality initiatives are probably needed to achieve larger improvements. Countries with several cycles of accreditation and other ongoing quality improvement strategies including disclosure about performance have been found to deliver a higher levels of recommended care over time [9, 49–51].

While the accreditation preparation process can be a critical step through which accreditation can have an impact, it can also pose some challenges. Experiences related to the process has been investigated in a number of studies [52–54]. A Danish study, interviewing staff from Danish public hospitals, found that the implementation process, especially in relation to the first accreditation cycle, was chaotic and characterized by uncertainly. Moreover, staff experienced being imposed to heavy administrative workloads of which the main task was to development and implement new guidelines [52]. In relation to the Faroe Islands first accreditation cycle, not all patients appeared to benefit from accreditation which may be explained the Faroese implementation process. The hospital had never prior participated in any systematic quality improvement activities and staff had to develop and implement of a large share of new guidelines, and at the same time monitor and study changes and act if the quality of care was considered inadequate. The heavy workload and new tasks may have been a contributing factor to the lack of consistent improvements across all diagnostic groups. Previous studies have reported that the implementation process is a period with less time and focus on patient care and many preparations may be performed at the expense of other tasks [52–54]. Moreover, the process may include unnecessary documentation and bureaucracy resulting in lower quality of services [54]. Although there are no detailed descriptions on how an accreditation model should be implemented to make the task worthwhile, there is some evidence that the process should be meaningful to the people in charge of the implementation [52]. A Canadian study found that the process become easier over time and the greatest benefits was related to second to fourth accreditation cycle. After 10 years of accreditation is likely to be a less challenging task [55].

As to the strengths to this study, firstly, the process performance measures used to collect data were developed by expert groups with extensive knowledge of the clinical conditions. Secondly, all the data were collected in relation to the hospitals’ first-time accreditation and therefore created a benchmark. Additionally, all data were collected by Faroese medical students with a local contextual knowledge. One of the medical students participated in both data collections to ensure uniformity. To ensure objectivity, data collections and analyses was performed by different people. These factors minimize the risk of information bias. However, we cannot exclude the risk of information bias. If changes in documentation practices occurred between the pre-and post-accreditation period, this could potentially have biased our analyses. Also, patients exposed to low quality of care could at least in theory also have been exposed to deficient documentation practices, which could have made it difficult for the data collectors to find the necessary information in the medical record and therefore to include the patient. In such cases, this could have led us to potentially overestimate the effect of accreditation, as the observed change in quality of care after accreditation could also reflect a change in documentation practices. Accreditation speaks to a systematic improvement of many workflows so we cannot dismiss information bias, although there is no immediate evidence to suggest this, as far more records were excluded in the patient inclusion process after accreditation due to errors compared with the period before accreditation.

The limitations included a moderate statistical precision and lack of an external control group. We aimed to include 601 patient records both before and after the accreditation. Yet due to many childbirths delivered without need of epidural or spinal block and/or acute cesarean section, we did not include the planned number of patients and it was not possible for legal and administrative reasons to compensate for the larger than expected number of patients without relevant process performance measures. The small sample size could potentially limit the generalizability of the study results. The risk of selection bias was likely small in this study as the included patients represent a random sample both before and after accreditation. Furthermore, the preparation of the list of patients for possible inclusion was performed by an administrative employee of the National Hospital who was not affiliated with the project and did not know the purpose of the research project. Before the patient sample was presented to the data collectors, patients’ appearance on the list was reassigned using the random function in excel, making sure that all patients had the same chance of being included. The risk of confounding is, as always in observational studies, also a possible cause of concern. However, we addressed the potential confounding by conducting stratified analyzes for each clinical condition as well as for individual process performance measures. Highly specific in- and exclusion criteria for each clinical condition and the included process performance measures ensured the eligibility of the patients and thereby comparability of the clinical needs in all analyzes. Adjusting for confounding factors would thus not give a reflection of true differences in the quality of care according to the definition of the performance measures but could potentially mask such differences. As this study did not include a control group, we cannot be sure that the changes in recommended care can be attributed to the first-time accreditation itself. However, the hospitals had not before or during the implementation of the accreditation model been subjected to any kind of systematic quality improvement initiatives or large structural changes. Thus, it is theoretically safe to assume, that the intervention contributed to the changes in recommended care. Additional support for this hypothesis could potentially have been obtained if a more systematic monitoring of the quality of care had been performed during the accreditation process rather than just the before and after assessment. Finally, the risk of chance findings should be considered as the statistical precision was modest in some of the analyzed subgroups. We did not correct for multiple testing as it is not routinely recommended as it will lead to fewer errors of interpretation when the data under evaluation are not random numbers but actual observations on nature [56]. Moreover, the study hypotheses are mutually supportive if results are pointing in the same direction, thus, allowing us to observe an overall pattern.

The results from this study contribute to the sparse knowledge about the association between accreditation and the delivery of recommended care in hospitals. Whilst accreditation is an externally driven compliance activity and therefore not necessarily focused on bottom-up quality improvements, our results show that it can impact on the level of evidence-based and guideline adherent care delivered to patients. In terms of generalizability and transferability, the results from this study can be understood and transferred to patients and hospitals elsewhere. All patients included were treated for common clinical conditions in hospital settings very similar to hospitals in other high-income countries. However, it is conceivable that transferability is strongest to healthcare systems that have not completed several rounds of accreditation and participated in years of systematic quality improvement activities. The fact that we did not find stronger associations between accreditation and the delivery of recommended care, could partly be explained by the Islands’ early stage of a quality improvement culture. First-time accreditation in the Faroe Islands has most likely affected many other areas of care than addressed in the current study. Further research is recommended to determine the impact of accreditation on other clinical conditions, patients’ outcomes and in different contexts.

## Conclusion

Accreditation was found to be associated with the delivery of more recommended care in hospitals never previously exposed to systematic quality and safety initiatives including accreditation. Especially patients with COPD, received significantly more recommended care after accreditation. However, the overall improvement of process performance measures was modest.

## Supplementary Information

**Additional file 1.**

**Additional file 2.**

## Data Availability

The datasets generated and analyzed during the current study are not publicly available due to the privacy of the individuals that participated in the study. According to Danish law, access to data can only be granted by applying the National hospital in the Faroe Islands, the Danish Data Protection Agency, the Faroese Data Protection Agency and the Danish Patient Safety Authority.

## Abbreviations

COPD: Chronic Obstructive Pulmonary Disease
RR: Relative Risk
IKAS: The Danish Institute for Quality and Accreditation in Healthcare
DDKM: The Danish Healthcare Quality program
RKKP: The Danish Clinical Quality Program – National Clinical Registries
TIA: Transient Ischemic Attack
RD: Risk Difference

## References

[1] Mainz J, Hansen AM, Palshof T, Bartels PD (2009). National quality measurement using clinical indicators: the Danish National Indicator Project. J Surg Oncol.

[2] Mainz J, Kristensen S, Bartels P (2015). Quality improvement and accountability in the Danish health care system. Int J Qual Health Care.

[3] White CM, Thomson JE, Statile AM, Auger KA, Unaka N, Carroll M, Tucker K, Fletcher D, Hall DE, Simmons JM (2017). Development of a new care model for hospitalized children with medical complexity. Hosp Pediatr.

[4] Grol R (2001). Successes and failures in the implementation of evidence-based guidelines for clinical practice. Med Care.

[5] Sungkar Y, Considine J, Hutchinson A (2018). Implementation of guidelines for sepsis management in emergency departments: a systematic review. Australas Emerg Care.

[6] Bernstein J (2004). Evidence-based medicine. J Am Acad Orthop Surg.

[7] Pronovost PJ, Berenholtz SM, Goeschel C, Thom I, Watson SR, Holzmueller CG, Lyon JS, Lubomski LH, Thompson DA, Needham D (2008). Improving patient safety in intensive care units in Michigan. J Crit Care.

[8] Siegfried A, Heffernan M, Kennedy M, Meit M (2018). Quality improvement and performance management benefits of public health accreditation: National Evaluation Findings. J Public Health Manag Pract.

[9] Falstie-Jensen AM, Bogh SB, Hollnagel E, Johnsen SP (2017). Compliance with accreditation and recommended hospital care-a Danish nationwide population-based study. Int J Qual Health Care.

[10] Falstie-Jensen AM, Larsson H, Hollnagel E, Norgaard M, Svendsen ML, Johnsen SP (2015). Compliance with hospital accreditation and patient mortality: a Danish nationwide population-based study. Int J Qual Health Care.

[11] Pronovost PJ, Watson SR, Goeschel CA, Hyzy RC, Berenholtz SM (2016). Sustaining reductions in central line-associated bloodstream infections in Michigan intensive care units: a 10-year analysis. Am J Med Qual.

[12] Patterson CH (1995). Joint commission on accreditation of healthcare organizations. Infect Control Hosp Epidemiol.

[13] Roberts JS, Coale JG, Redman RR (1987). A history of the joint commission on accreditation of hospitals. JAMA.

[14] Joint Commission International. https://www.jointcommissioninternational.org/. Accessed 24 June 2021.

[15] Flodgren G, Goncalves-Bradley DC, Pomey MP (2016). External inspection of compliance with standards for improved healthcare outcomes. Cochrane Database Syst Rev.

[16] Hinchcliff R, Greenfield D, Moldovan M, Westbrook JI, Pawsey M, Mumford V, Braithwaite J (2012). Narrative synthesis of health service accreditation literature. BMJ Qual Saf.

[17] Bogh SB, Falstie-Jensen AM, Bartels P, Hollnagel E, Johnsen SP (2015). Accreditation and improvement in process quality of care: a nationwide study. Int J Qual Health Care.

[18] Alkhenizan A, Shaw C (2011). Impact of accreditation on the quality of healthcare services: a systematic review of the literature. Ann Saudi Med.

[19] Greenfield D, Braithwaite J (2008). Health sector accreditation research: a systematic review. Int J Qual Health Care.

[20] Brubakk K, Vist GE, Bukholm G, Barach P, Tjomsland O (2015). A systematic review of hospital accreditation: the challenges of measuring complex intervention effects. BMC Health Serv Res.

[21] Shaw CD, Groene O, Botje D, Sunol R, Kutryba B, Klazinga N, Bruneau C, Hammer A, Wang A, Arah OA (2014). The effect of certification and accreditation on quality management in 4 clinical services in 73 European hospitals. Int J Qual Health Care.

[22] Mumford V, Forde K, Greenfield D, Hinchcliff R, Braithwaite J (2013). Health services accreditation: what is the evidence that the benefits justify the costs?. Int J Qual Health Care.

[23] Mumford V, Greenfield D, Hogden A, Forde K, Westbrook J, Braithwaite J (2015). Counting the costs of accreditation in acute care: an activity-based costing approach. BMJ Open.

[24] Rockwell DA, Pelletier LR, Donnelly W (1993). The cost of accreditation: one hospital's experience. Hosp Community Psychiatry.

[25] World Bank Open Data. https://data.worldbank.org/country/faroe-islands. Accessed 24 June 2021.

[26] Statistics Faroe Islands. https://hagstova.fo/en/population/population/population. Accessed 24 June 2021.

[27] Introduction to DDKM. https://www.ikas.dk/den-danske-kvalitetsmodel/ddkm-in-english/introduction-to-ddkm/. Accessed 24 June 2021.

[28] Akkrediteringsstandarder for de færøske sygehuse. https://www.ikas.dk/deltagere-i-ddkm/faeroeske-sygehuse/. Accessed 24 June 2021.

[29] Regionernes kliniske kvalitetsudviklingsprogram. https://www.rkkp.dk/inenglish/. Accessed 24 June 2021.

[30] Johnsen SP, Ingeman A, Hundborg HH, Schaarup SZ, Gyllenborg J (2016). The Danish stroke registry. Clin Epidemiol.

[31] Lange P, Tøttenborg SS, Sorknæs AD, Andersen JS, Søgaard M, Nielsen H, Thomsen RW, Nielsen KA (2016). Danish register of chronic obstructive pulmonary disease. Clin Epidemiol.

[32] Jørgensen ME, Kristensen JK, Reventlov Husted G, Cerqueira C, Rossing P (2016). The Danish adult diabetes registry. Clin Epidemiol.

[33] Schjødt I, Nakano A, Egstrup K, Cerqueira C (2016). The Danish heart failure registry. Clin Epidemiol.

[34] Bliddal M, Broe A, Pottegård A, Olsen J, Langhoff-Roos J (2018). The Danish medical birth register. Eur J Epidemiol.

[35] REDCap. https://projectredcap.org/. Accessed 24 June 2021.

[36] McHugh ML (2012). Interrater reliability: the kappa statistic. Biochem Med (Zagreb).

[37] Shwartz M, Restuccia JD, Rosen AK (2015). Composite measures of health care provider performance: a description of approaches. Milbank Q.

[38] Lutfiyya MN, Sikka A, Mehta S, Lipsky MS (2009). Comparison of US accredited and non-accredited rural critical access hospitals. Int J Qual Health Care.

[39] Woolf SH, Grol R, Hutchinson A, Eccles M, Grimshaw J (1999). Clinical guidelines: potential benefits, limitations, and harms of clinical guidelines. BMJ.

[40] Bogh SB, Falstie-Jensen AM, Hollnagel E, Holst R, Braithwaite J, Johnsen SP (2016). Improvement in quality of hospital care during accreditation: a nationwide stepped-wedge study. Int J Qual Health Care.

[41] Devkaran S, O'Farrell PN (2015). The impact of hospital accreditation on quality measures: an interrupted time series analysis. BMC Health Serv Res.

[42] Bogh SB, Falstie-Jensen AM, Hollnagel E, Holst R, Braithwaite J, Raben DC, Johnsen SP (2017). Predictors of the effectiveness of accreditation on hospital performance: a nationwide stepped-wedge study. Int J Qual Health Care.

[43] Lokke A, Hilberg O, Kjellberg J, Ibsen R, Jennum P (2014). Economic and health consequences of COPD patients and their spouses in Denmark--1998-2010. COPD.

[44] Tottenborg SS, Thomsen RW, Nielsen H, Johnsen SP, Frausing Hansen E, Lange P (2013). Improving quality of care among COPD outpatients in Denmark 2008-2011. Clin Respir J.

[45] Løkke A, Hilberg O, Tønnesen P, Ibsen R, Kjellberg J, Jennum P (2014). Direct and indirect economic and health consequences of COPD in Denmark: a national register-based study: 1998-2010. BMJ Open.

[46] Nolan T, Berwick DM (2006). All-or-none measurement raises the bar on performance. JAMA.

[47] Ingeman A, Pedersen L, Hundborg HH, Petersen P, Zielke S, Mainz J, Bartels P, Johnsen SP (2008). Quality of care and mortality among patients with stroke: a nationwide follow-up study. Med Care.

[48] Nielsen KA, Jensen NC, Jensen CM, Thomsen M, Pedersen L, Johnsen SP, Ingeman A, Bartels PD, Thomsen RW (2009). Quality of care and 30 day mortality among patients with hip fractures: a nationwide cohort study. BMC Health Serv Res.

[49] McGlynn EA, Asch SM, Adams J, Keesey J, Hicks J, DeCristofaro A, Kerr EA (2003). The quality of health care delivered to adults in the United States. N Engl J Med.

[50] Runciman WB, Hunt TD, Hannaford NA, Hibbert PD, Westbrook JI, Coiera EW, Day RO, Hindmarsh DM, McGlynn EA, Braithwaite J (2012). CareTrack: assessing the appropriateness of health care delivery in Australia. Med J Aust.

[51] Braithwaite J, Hibbert PD, Jaffe A, White L, Cowell CT, Harris MF, Runciman WB, Hallahan AR, Wheaton G, Williams HM (2018). Quality of health Care for Children in Australia, 2012-2013. JAMA.

[52] Bogh SB, Blom A, Raben DC, Braithwaite J, Thude B, Hollnagel E, Plessen CV (2018). Hospital accreditation: staff experiences and perceptions. Int J Health Care Qual Assur.

[53] Chiu A, Seto WH, Lai L (2011). Journey of a Hong Kong public teaching hospital in preparation of hospital accreditation. Hong Kong Med J.

[54] Vali L, Mehrolhasani MH, Mirzaei S, Oroomiei N (2020). Challenges of implementing the accreditation model in military and university hospitals in Iran: a qualitative study. BMC Health Serv Res.

[55] Pomey MP, Lemieux-Charles L, Champagne F, Angus D, Shabah A, Contandriopoulos AP (2010). Does accreditation stimulate change? A study of the impact of the accreditation process on Canadian healthcare organizations. Implement Sci.

[56] Rothman KJ (1990). No adjustments are needed for multiple comparisons. Epidemiology.

