# Supplementary material for: The association between first-time accreditation and the delivery of recommended care: a before and after study in the Faroe Islands
Source: BMC Health Serv Res. 2021 Sep 5;21:917. doi: 10.1186/s12913-021-06952-w (PMC8418753; doi:10.1186/s12913-021-06952-w)
Supplement: Supplementary file 1 — Additional file 1. [file 12913_2021_6952_MOESM1_ESM.pdf]

**Additional file 1.** Process performance measures, time frame criteria and diagnostic codes (ICD-10, version 2016)

| **Process performance measures (diagnosis codes)** | **Time frame criteria** |
|---|---|
| **Stroke and TIA** (I61, I63, I64, G45) | |
| Door-to-needle time, thrombolysis treatment for acute ischemic stroke | 1 hour after arrival to the thrombolysis unit |
| Admission to stroke unit, acute stroke | Second day of hospitalization |
| Oral antithrombotic therapy initiated:<br><br>Antiplatelet therapy for acute ischemic stroke without atrial fibrillation<br><br>Antiplatelet therapy for TIA without atrial fibrillation<br><br>Oral anticoagulant therapy for acute ischemic stroke with atrial fibrillation<br><br>Oral anticoagulant therapy for TIA with atrial fibrillation | <br><br>Second day of hospitalization<br><br>Second day after first contact with a hospital<br><br>14$^{th}$ day of hospitalization<br><br>14$^{th}$ day after first contact with a hospital |
| Examination, CT/MR scan:<br><br>Acute stroke<br><br>TIA | <br><br>6 hours after hospitalization<br><br>6 hours after first contact with a hospital |
| Assessment by a physiotherapist, acute stroke | Second day of hospitalization |
| Assessment by an occupational therapist, acute stroke | Second day of hospitalization |
| Early mobilization, acute stroke | First day of hospitalization |
| Nutritional risk assessment, acute stroke | Second day of hospitalization |
| Screening for dysphagia, acute stroke. Indirect test | First day of hospitalization |

| | |
|---|---|
| Screening for dysphagia, acute stroke. Direct test | First day of hospitalization |
| Examination of carotid arteries with ultrasound CT-/MR angiography:<br><br>Acute ischemic stroke<br><br>TIA | <br><br>4th day of hospitalization<br><br>4th day of hospitalization |
| **Bleeding gastric ulcer** (K25.0, K25.4, K26.0, K26.4, K27.0, K27.4) | |
| Treatment of affected circulation | ≤ 60 minutes |
| Endoscopy:<br><br>1.Patient just arrived at the hospital<br><br>2.Hospitalized patient | <br><br>≤ two hours after arrival at the hospital<br><br>≤ two hours after examination by surgeon |
| Directly transferred to an operating/endoscopy theater due to sustained affected circulation | Immediately |
| Restrictive blood transfusion therapy | Hemoglobin ≥ 4.3 mM |
| Risk stratification, Rockall score | Before the end of the gastroscopy |
| Treatment with adrenaline-saline injection and another hemostatic modality | During endoscopy |
| Treatment with enteral or intravenous proton pump inhibitor | During hospitalization |
| Test for Helicobacter Pylori | During hospitalization or a scheduled test<br><br>after discharge |
| **Diabetes** (E10 - E10.9, E11 - E11.9, E13 - E13.9, E14 - E14.9) | |
| Antidiabetic treatment initiated, Patients (Type 2 diabetes) with a HbA1c ≥ 75 mmol/mol | Every year |

| | |
|---|---|
| Blood pressure control | Every year |
| Antihypertensive treatment initiated, Blood pressure > 140/90 | Every year |
| LDL cholesterol control, Patients ≥ 30 years | Every second year |
| Lipid lowering treatment initiated, Patients (Type 2 diabetes) ≥ 40 years with LDL cholesterol > 2.5 | Every year |
| Albuminuria control | Every second year |
| Treatment with ACE inhibitor / ATII receptor antagonist, Patients with micro- or macroalbuminuria | Every year |
| Ophthalmological examination | Every second year |
| Ophthalmological examination | Every fourth year |
| Feet examination | Every second year |
| Smoking status | Every year |
| Call for smoking cessation, Patients who are smoking or recently stopped smoking | Every year |
| **Chronic obstructive pulmonary disease (COPD)**<br>(Outpatient: J44 or J96 including secondary diagnosis J44)<br>(Inpatient: J44 or J96, J13 - J18 including secondary diagnosis J44) | |
| Measured and recorded $FEV_1$, Outpatient | Every year |
| Calculated and recorded Body Mass Index, Outpatient | Every year |
| Measured and recorded shortness of breath using the MRC scale, Outpatient | Every year |
| Queried and recorded smoking status, Outpatient | Every year |
| Offered to participate in pulmonary rehabilitation, Patients with MRC-level ≥ 3, Outpatient | Every second year |

| | |
|---|---|
| Implementation of 50 % of the pulmonary rehabilitation in the hospital, Outpatient | Every year |
| Treatment with long-term inhaled bronchodilator either as LAMA or LABA, Patients with MRC level ≥ 2, Outpatient | Every year |
| Treatment with ICS, Patients with MRC level ≥ 2, treated with long-term inhaled bronchodilator, $FEV_1$ < 50 % of expected value, Outpatient | Every year |
| Inhalation technique control, Patients treated with inhalation medicine, Outpatient | Every year |
| Registration of acute exacerbations pr. year, Outpatient | Every year |
| Treatment with assisted ventilation (NIV), Hospitalized patients with acute exacerbation, Inpatient | Every year |
| **Childbirth** (O80.0 - O84.9) | |
| Construction of epidural or spinal block for birth | ≤ 1 hour from ordering |
| Emergency caesarean section grade 1 | Baby born < 15 minutes from ordering |
| Emergency caesarean section grade 2 | Baby born < 30 minutes from ordering |
| **Heart failure** (I11.0, I13.0, I13.2, I42.0, I42.6, I42.7, I42.9, I50.0, I50.1, I50.9) | |
| Examination, Echocardiography | 6 months before and no later than 7 weekdays after admission/initiation of outpatient treatment |
| NYHA classification | At the first discharge/first outpatient visit or within the first 12 weeks of their illness |

| | |
|---|---|
| Treatment with ACE-inhibitor/ATII-receptor antagonist, Patients with a reduced systolic function LVEF ≤ 40% | Start treatment no later than 8 weeks after admission/first outpatient visit |
| Treatment with Beta blocker, Patients with LVEF ≤ 40% | Start treatment no later than 12 weeks after admission/first outpatient visit |
| Treatment with Aldosterone antagonist, Patients with LVEF ≤ 35% | Start treatment no later than 12 weeks after admission/first outpatient visit |
| Individualized supervised exercise training by physiotherapist in the hospital, or referred to training in a community setting, Patients with LVEF ≤ 40% | No later than 12 weeks after admission/initiation of outpatient treatment |
| Initiate an individualized patient education during follow-up in a heart failure clinic | No later than 12 weeks after admission/initiation of outpatient treatment |
| **Hip Fracture** (S72.0, S72.1, S72.2) | |
| Pre-operative optimization plan performed by specialist or doctor in specialist training | ≤ 4 hours after arriving at the hospital |
| Operation | ≤ 24 hours after arriving at the hospital |
| Operation | ≤ 36 hours after arriving at the hospital |
| Mobilization after surgery | ≤ 24 hours after surgery |
| Assessment and reporting basic mobility score with Cumulated Ambulation Score | Prior to the actual fracture |
| Assessment and reporting basic mobility score with Cumulated Ambulation Score | Before discharge |
| Preparation of nutrition plan | During hospitalization |
| Assessment of medical osteoporosis prophylaxis and treatment with calcium and vitamin D | During hospitalization |

| | |
|---|---|
| Falling prophylaxis | During hospitalization |
| Preparation of a rehabilitation plan with an ADL function assessment prior to the bone fracture and an assessment before the discharge | Before discharge |
