# Supplementary material for: The association between first-time accreditation and the delivery of recommended care: a before and after study in the Faroe Islands
Source: BMC Health Serv Res. 2021 Sep 5;21:917. doi: 10.1186/s12913-021-06952-w (PMC8418753; doi:10.1186/s12913-021-06952-w)
Supplement: Supplementary file 2 — Additional file 2. [file 12913_2021_6952_MOESM2_ESM.pdf]

Additional file 2. The proportion of patient pathways who received 100% of the recommended care before and after the first hospital accreditation

	Before Accreditation 2012 and 2013		After Accreditation 2017 and 2018			
	All recommended care (N)⋆	All recommended care (%)	All recommended care (N)	All recommended care (%)	RR¶ (95% CI)†	RD§ (95% CI)
Clinical condition						
Stroke and TIA	2/27	7.4	17/62	27.4	3.69 (0.76;17.91)	0.20 (-0.01;0.41)
Bleeding gastric ulcer	0/12	0.0	0/3	0.0	-	-
Diabetes	17/219	7.8	3/37	8.1	1.04 (0.84;1.29)	0.003 (-0.013;0.019)
COPD	6/174	3.5	9/108	8.3	2.41 (1.31;4.46)	0.05 (0.01;0.09)
Childbirth	1/13	7.7	9/45	20.0	2.59 (1.93;3.49)	0.12 (0.09;0.15)
Heart failure	5/23	21.7	5/52	9.6	0.44 (0.29;0.66)	-0.12 (-0.25;0.01)
Hip fracture	0/68	0.0	0/24	0.0	-	-
Total all-or-none	31/536	5.8	43/331	13.0	2.32 (2.31;2.34)	0.08* (0.04;0.11)

⋆Number of patient pathways who received 100% of the recommended care according to the clinical condition divided by the number of patient pathways eligible for the care. No restrictions to the number of relevant process performance measures. ¶RR, Relative Risk. Adjusted for dependence between observations at patient level and cluster effect at hospital level. †CI, confidence interval. §RD, Risk difference. Adjusted for cluster effect at hospital level. *Adjusted for dependence between observations at patient level and cluster effect at hospital level.
